# Effect of Nickel and Titanium on Properties of Fe-Al-Si Alloy Prepared by Mechanical Alloying and Spark Plasma Sintering

**DOI:** 10.3390/ma13030800

**Published:** 2020-02-10

**Authors:** Pavel Novák, Zdeněk Barták, Kateřina Nová, Filip Průša

**Affiliations:** 1 Department of Metals and Corrosion Engineering, University of Chemistry and Technology, Technická 5, 166 28 Prague 6, Czech Republic; bartak@svuom.cz (Z.B.); novakx@vscht.cz (K.N.); prusaf@vscht.cz (F.P.); 2 SVÚOM s.r.o., U Měšťanského pivovaru 934/4, 170 00 Prague 7, Holešovice, Czech Republic

**Keywords:** Fe-Al-Si alloy, nickel, titanium, oxidation, wear resistance

## Abstract

This paper describes the structure and properties of an innovative Fe-Al-Si alloy with a reduced amount of silicon (5 wt. %) in order to avoid excessive brittleness. The alloy was produced by a combination of mechanical alloying and spark plasma sintering. Nickel and titanium were independently tested as the alloying elements for this alloy. It was found that wear resistance, which reached values comparable with tool steels, could be further improved by the addition of nickel. Nickel also improved the high-temperature oxidation behavior, because it lowers the liability of the oxide layers to spallation. Both nickel and titanium increased the hardness of the alloy. Titanium negatively influenced oxidation behavior and wear resistance because of the presence of titanium dioxide in the oxide layer and the brittle silicides that caused chipping wear, respectively.

## 1. Introduction

Iron–aluminum alloys have been investigated since 1894, when the positive effect of aluminum addition on the high-temperature oxidation of iron was reported [1]. This effect is caused by the formation of protective layer of aluminum oxide, as reported later [2]. In an Fe-Al system, a series of intermetallics have been described, namely Fe_3_Al, FeAl, Fe_2_Al_5_ and FeAl_3_ (also mentioned as Al_13_Fe_4_) [2,3,4]. The first two ones, which are in fact ordered solid solutions, have gained technical importance. Materials based on these phases have been thoroughly and successfully tested for oxidation resistance in the air, as well as sulphur-containing environments, carbon dioxide with water vapor, salts, electrolytes, and even the glass melts [2,5,6,7,8,9,10]. The special “Exo-Melt” process [11] and casting have been proposed as the manufacturing route of such materials. Special carbon-containing Fe-Al-based alloys were developed in the 1950’s in order to ensure good casting properties [1]. These alloys were composed of an FeAl phase and aluminum carbide (Al_4_C_3_) or mixed aluminum–iron carbide (Fe_3_AlC) with perovskite structure [12,13]. Aluminum carbide, even though it is a hard phase with a reinforcing effect, brings a big problem to these materials, because it hydrolyses to methane when it gets into the contact with acids or hot water vapor [14], which could lead to the damage of the material.

Therefore, our team focused on the development of carbon-free iron aluminide-based materials. It has been proven that silicon positively affects oxidation resistance [15]. Recently, we described the microstructure, phase composition, oxidation behavior, and mechanical properties of an FeAl20Si20 alloy (in wt. %) that was easily producible by various powder metallurgical processes, such as self-propagating high-temperature synthesis [15] and mechanical alloying in combination with spark plasma sintering [16]. The alloys exhibited a very good oxidation resistance at high temperatures—much better than binary Fe-Al and Fe-Si alloys [17]. The improvement of oxidation resistance does not lie in the incorporation of silicon to the oxide layer in a significant amount; rather, it lies in the formation of large volume fraction of silicides under the oxide layer, when aluminum diffuse to the surface in order to form Al_2_O_3_. Additionally, it has been found that the presence of silicon reduces the amount of iron oxide in scales, causing their better adherence to a substrate due to a more favorable Pilling–Bedworth ratio [16]. However, the FeAl20Si20 alloy is very brittle. Powder metallurgy methods, including our high-energy mechanical alloying [18] and spark plasma sintering, allowed for an increase in the fracture toughness, but the values at room temperature still reached the parameters of brittle ceramics, i.e., approximately 3.5 MPa.m^1/2^ [16]. Due to these parameters, the alloy could be applicable as a protective coating rather than as a bulk material.

In recent research, we studied the high-temperature oxidation resistance of Fe-Al-Si alloys and its dependence on the Al:Si ratio, and we found that the 35:5 provided almost the same oxidation performance [17]. Since silicon is listed as a critical raw material in the EU [19], the minimization of its amount is reasonable. In a parallel research, our team studied Ti-Al alloys and proved that silicon also improves their oxidation behavior and reinforces the material by forming hard Ti_5_Si_3_ silicides [20]. On the other hand, nickel is known to form stable aluminides rather than silicides [21].

Therefore, this work aimed at a possible improvement of the properties of a lower-silicon FeAl35Si5 alloy by the addition of titanium as the expected silicide-forming element and nickel as the probable aluminide stabilizer. The tests were intended to study the high temperature oxidation behavior, basic mechanical properties, and tribological properties.

## 2. Materials and Methods 

The alloys summarized in Table 1 were produced by mechanical alloying (MA) and subsequent spark plasma sintering (SPS). For mechanical alloying, the planetary ball mill (PM 100 CM, Retsch, Haan, Germany) and following conditions were utilized: a milling duration of 10 h, a change of rotation direction each 30 min, a rotational velocity of 400 rpm, an argon atmosphere, a powder batch of 20 g, and a ball-to-powder weight ratio of approximately 15:1. The blends for mechanical alloying were prepared from following elemental powders: iron (purity 99.9%, particle size < 44 μm, supplied by Strem Chemicals, Newburyport, MA, USA, aluminum (purity 99,7%, particle size < 44 μm, supplied by Strem Chemicals), silicon (purity 99.5%, particle size < 44 μm, supplied by Alfa Aesar, Haverhill, MA, USA), nickel (purity 99.99%, particle size < 150 μm, supplied by Strem Chemicals), and titanium (purity 99.5%, particle size < 100 μm, supplied by Strem Chemicals). The mechanically alloyed powders were consolidated by the SPS method by means of an HP D10 device (FCT Systeme, Rauenstein, Germany) by using a pressure of 48 MPa, a temperature of 1000 °C, a duration of 10 min, a heating rate of 300 K/min, and a cooling rate of 50 K/min in order to avoid the cracking of the product. The weight of the batch for sintering was approximately 5 g. The conditions of both MA and SPS were selected on the basis of our previous research [18,22]. The applied amounts of nickel and titanium originated from our previous research on Fe-Al-Si-X alloys that were prepared by reactive sintering [23].

The microstructure of the alloys that were produced by combination of mechanical alloying and spark plasma sintering was studied with a VHX 5000 digital microscope (Keyence, USA) after etching by modified Kroll’s reagent (5 mL of HNO_3_, 10 mL of HF, and 85 mL of H_2_O) and by a Lyra3 GMU scanning electron microscope (Tescan, Brno, Czech Republic) with an X-max 80 mm^2^ energy dispersive spectrometer (EDS) (Oxford Instruments, High Wycombe, UK) after etching in Keller’s reagent (2.5 mL of HNO_3_, 1 mL of HF, 1.5 mL of HCl, and 95 mL of H_2_O). Phase composition was identified by X-ray diffraction analysis (XRD) while using an X’Pert Pro 2.0a (PANalytical, Almelo, Netherlands) X-ray diffractometer. The crystallite size of the FeAl phase in the tested alloys was calculated by Sherrer’s method in the HighScore software package, which was also applied for the qualitative evaluation of XRD patterns while using a PDF-2 database. Sherrer’s method uses following formula:

(1) $$\tau =\frac{K\times \lambda }{\beta \times \cos\theta }$$

where *τ*, *K*, *λ*, *β* and *θ* are crystallite size (m), shape factor (used typical 0.9), wavelength (m), line broadening at half the maximum intensity (°), and Bragg angle (°), respectively.

The mechanical properties of the SPS-consolidated material were determined by the means of microhardness measurements. For this purpose, the Vickers method with a load of 9.8 N (HV1) was applied. The wear resistance was measured by using the TriboTester ball-on-disc tribometer (Tribotechnic, Clichy, France) in the linear reciprocating mode (excenter of 5 mm), where the “ball” of 6 mm in diameter was made of alumina (α-Al_2_O_3_) and the “disc” was the sample polished to a roughness of approximately 0.005 µm. No lubrication was used. The normal force that was used in the test was 5 N, and the sliding distance was 20 m. The wear rate was calculated from the wear track section area by Equation (2):

(2) $$w=\frac{A\times e}{F\times l}$$

where *w*, *A*, *e*, *F* and *l* are wear rate (mm^3^N^−1^∙m^−1^), wear track section area (mm^2^), excenter (5 mm), normal force (5 N), and sliding distance (20 m), respectively. The wear track section area was measured by means of a skidless contact profilometer probe (Tribotechnic, Clichy, France). Wear tracks were observed by means of a scanning electron microscope (SEM) VEGA 3 (Tescan, Brno, Czech Republic) in the backscattered electrons (BSE) mode.

Cyclic oxidation tests were carried out at 800 °C in air with a cycle duration of 50 h. Samples were separately located in alumina crucibles during exposure. The samples were air-cooled and weighed by an analytical scale Pioneer Plus (Ohaus, USA) with an accuracy of 0.0001 g after each cycle. Oxidation rate kinetics was observed on the basis of a specific weigh gain, i.e., the increase of weight of a sample caused by the embedding of oxygen to the oxides formed on the surface divided by the exposed area of the sample’s surface. The delaminated oxides (oxides detached from the samples’ surface and left in the crucibles) were also weighed and evaluated. The microstructure and chemical composition of the oxide layers were documented by the SEM with an X-max 20 mm^2^ energy dispersive spectrometer (EDS) (Oxford Instruments, High Wycombe, UK), and phase composition was determined by XRD. For the observation, the secondary electrons (SE) mode was applied. Oxidation kinetics was evaluated by fitting the specific weight gains vs. the duration of oxidation by parabolic law; see Equation (2):

(3) $$k_{p}=\frac{{\left(\frac{\Delta m}{A}\right)}^{2}}{t}$$

where *k_p_*, Δ*m*, *A* and *t* are parabolic rate constant (g^2^∙m^−4^∙s^−1^), weight gain (g), exposed area (m^2^) and duration of oxidation (s), respectively.

## 3. Results

### 3.1. Microstructure and Phase Composition

**Figure 1:**
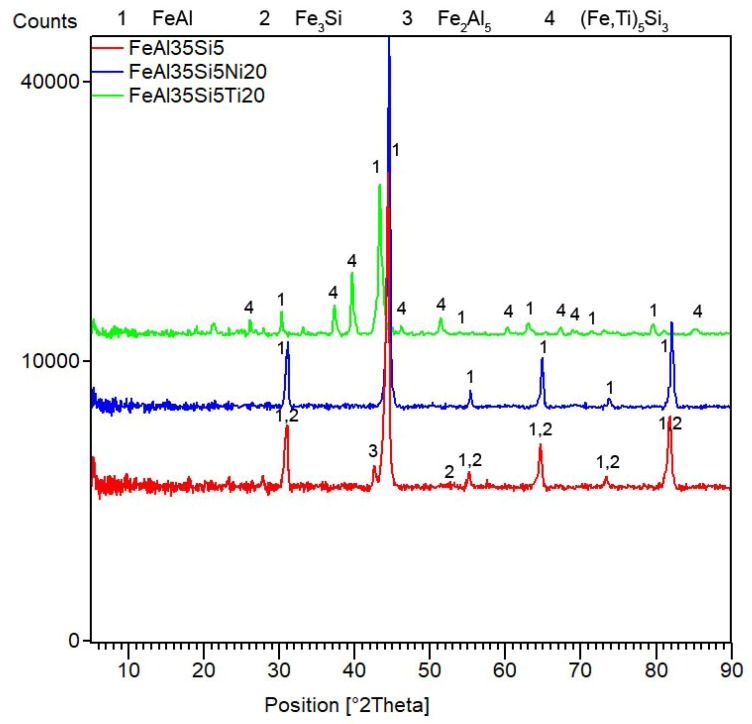
X-ray diffraction analysis (XRD) patterns of the tested bulk alloys that were produced by mechanical alloying and spark plasma sintering.

**Figure 2:**
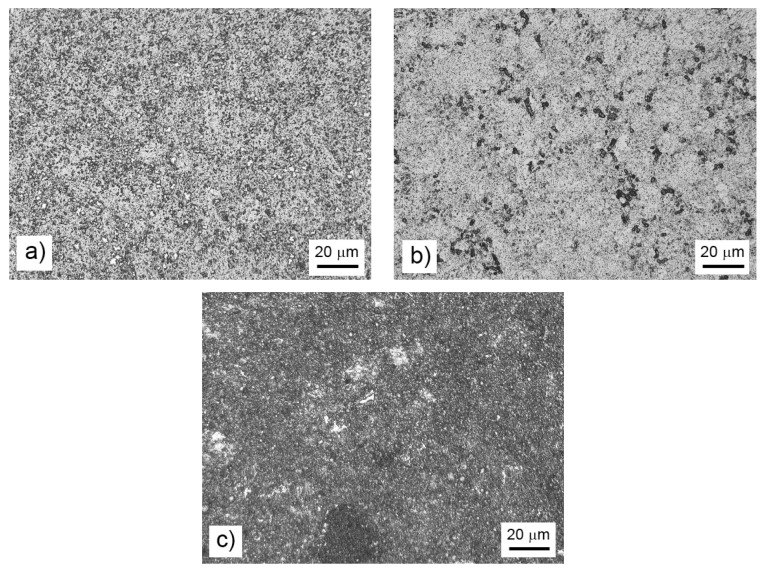
Microstructure of the bulk FeAl35Si5 alloy (**a**), the FeAl35Si5Ni20 alloy (**b**), and the FeAl35Si5Ti20 alloy (**c**) that were produced by mechanical alloying and spark plasma sintering.

**Figure 3:**
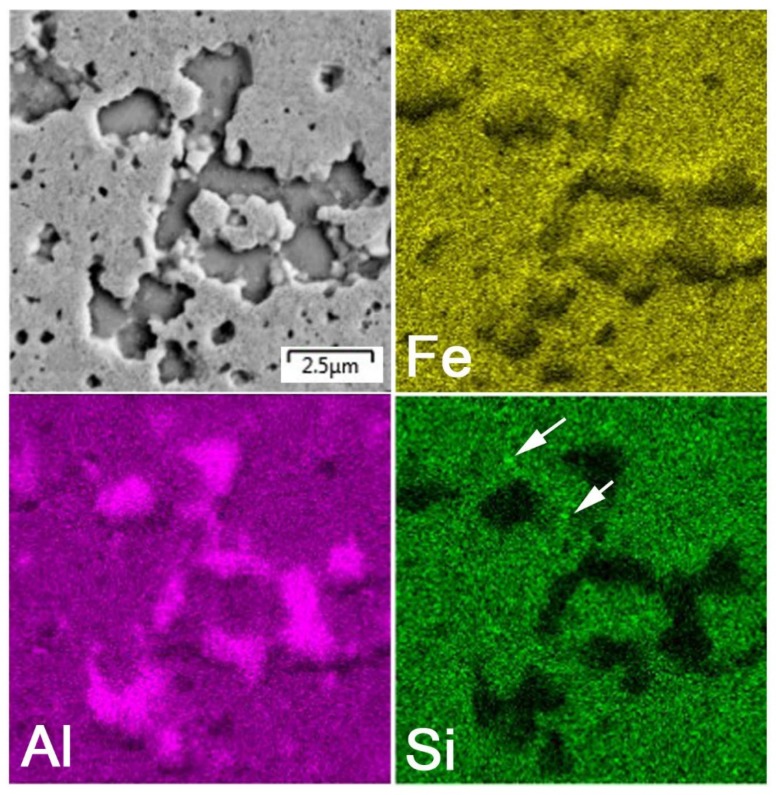
Energy dispersive spectrometer (EDS) elemental map of the bulk FeAl35Si5 alloy.

**Figure 4:**
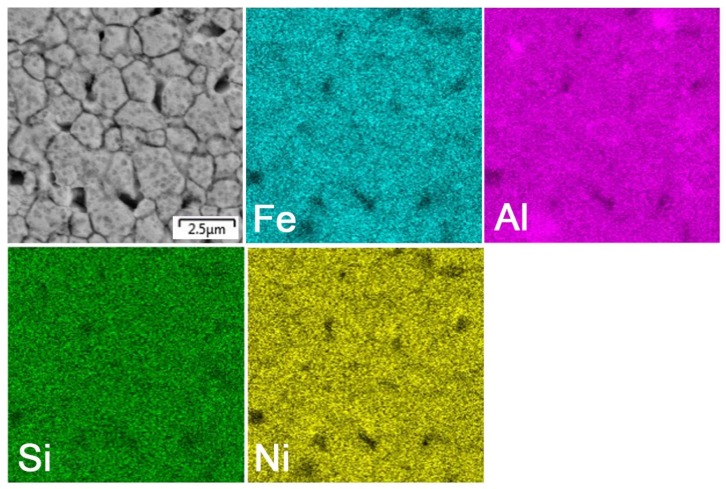
EDS elemental map of the bulk FeAl35Si5Ni20 alloy.

After mechanical alloying and spark plasma sintering, the FeAl35Si5 alloy was composed of the FeAl (B2 structure prototype, Pm-3m), Fe_3_Si (D0_3_ structure prototype, Fm-3m) and Fe_2_Al_5_ (Cmcm) phases, as proven by XRD (Figure 1). The recognition of the FeAl and Fe_3_Si phases by XRD is not fully reliable due to overlaps of most of the peaks of these phases. In addition, there were differences of the lattice parameters from the tabled ones due to a non-equilibrium nature of mechanical alloying. However, it was possible to recognize them by optical microscopy. On the optical micrographs, the silicides appear as the white particles, Fe_2_Al_5_ forms the dark grey regions, and the FeAl phase is the matrix (Figure 2a) due to different etching sensitivity of the phases [17]. These phases, especially the aluminum-rich Fe_2_Al_5_ phase, are visible on the EDS map (Figure 3). Iron silicides are very fine and hardly visible on the silicon distribution map (marked by the arrows in Figure 3). When nickel was added, the Fe_2_Al_5_ phase disappeared (Figure 1, Figure 2b and Figure 4). It could have a beneficial effect on fracture toughness, because the Fe_2_Al_5_ phase is known as highly brittle, just like Fe_3_Al_2_Si_3_ in a high-silicon FeAl20Si20 alloy, which was recently investigated [16]. According to the optical microscopy and EDS map (Figure 4), the silicides also completely disappeared when the nickel was added. This confirms the presumption that nickel would stabilize the FeAl aluminide phase, since the highly stable NiAl phase [21] had the same crystal structure (B2, Pm-3m) as the FeAl. Due to the elimination of the Fe_3_Si phase, the silicon was dissolved in the matrix. The distribution of nickel in the alloy was nearly homogeneous; see Figure 4. The nickel addition was found to increase the crystallite size of the FeAl phase and to decrease the interplanar distance of the (110) planes (Table 2).

**Figure 5:**
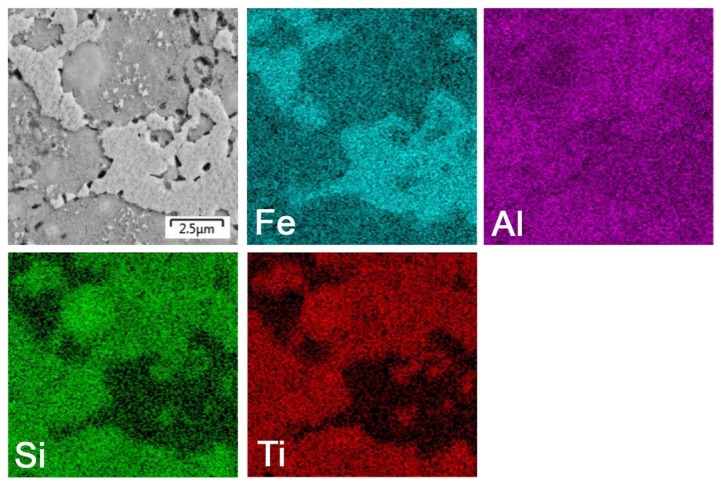
EDS elemental map of the bulk FeAl35Si5Ti20 alloy.

The titanium addition also destabilized Fe_2_Al_5_, which was not present in the titanium-containing alloy (Figure 1). The matrix of all titanium-containing alloys was still the FeAl phase, but titanium also formed a new phase (Figure 1)—(Fe,Ti)_5_Si_3_ silicide (P63/mmc). The presence of (Fe,Ti)_5_Si_3_ particles is reflected by the fine white particles present in Figure 2c and is also reflected on the EDS map in Figure 5. The diffraction lines of the FeAl phase were systematically and strongly shifted to lower diffraction angles, i.e., higher interplanar distances (Figure 1, Table 2), indicating the probably partial substitution of iron by titanium, which has higher atomic radius (176 pm) than iron (156 pm).

### 3.2. Mechanical a Tribological Properties

The hardness of the FeAl35Si5 base alloy reached 819 ± 20 HV1 (Table 3). The addition of nickel increased the hardness to 914 ± 19 HV1, even though there were no hard phases like Fe_2_Al_5_ and silicides (Figure 1 and Figure 2b). The explanation for this probably lies in the solution strengthening of iron aluminide by nickel and dissolved silicon, as discussed above. The titanium addition allowed the hardness to reach a higher value of 963 ± 13 HV1, probably mainly due to the presence of a hard (Fe,Ti)_5_Si_3_ phase. The hardness of Ti_5_Si_3_ has been previously determined as approximately 1500 HV0.005 [24].

In many cases, the high hardness also implies a high wear resistance. However, wear rate is influenced not only by hardness but also by the toughness and sliding properties of the material [25]. In this particular case, the wear rate also did not fully reflect the hardness trends. The wear rate was found to decrease with the addition of nickel, but it strongly increased to a value that was more than two times higher in the case of the titanium addition (Table 3). The friction coefficient showed the same general trends as the wear rate. The lowest friction coefficient was measured in the case of the nickel-alloyed materials, and the highest one was measured for the titanium-containing alloy (Table 3).

**Figure 6:**
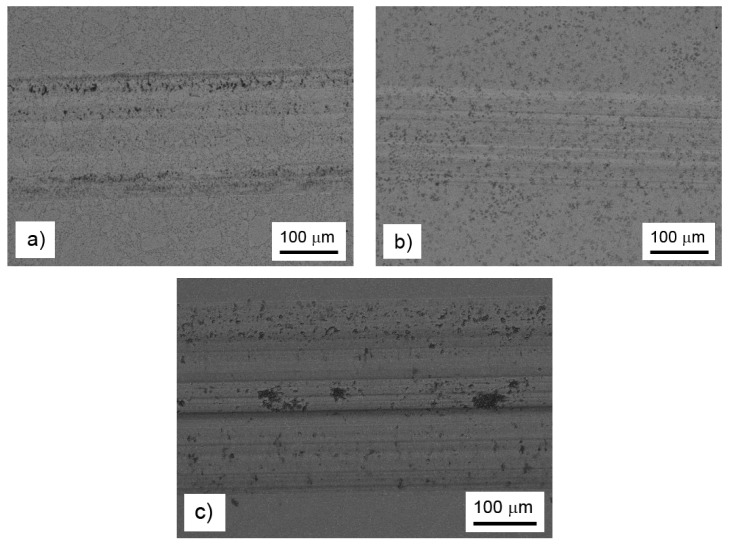
Wear tracks on the FeAl35Si5 alloy (**a**), the FeAl35Si5Ni20 alloy (**b**), and the FeAl35Si5Ti20 alloy (**c**), as documented by backscattered electrons (BSE)-SEM.

In order to explain the observed influence of the alloying elements on the wear resistance and friction coefficient, the morphology of the wear tracks was observed by BSE-SEM (Figure 6). In the case of the FeAl35Si5 base alloy, the wear track contained traces of abrasive wear (longitudinal scratches), traces of small particles’ chipping (black regions in the wear track), and minor signs of the oxidized wear debris (appears darker due to the presence of oxygen with lower proton number than the other elements in the material) at the sides of the track. The morphology of the removed particles corresponded to the Fe_2_Al_5_ phase in Figure 2a. On the other hand, there were absolutely no signs of chipping wear or visible oxidation in the nickel-alloyed material (Figure 6b). The wear was purely abrasive, as seen from the scratches. The absence of the chipping was probably caused by the elimination of the brittle particles of Fe_2_Al_5_ and silicides due to the addition of nickel (Figure 1 and Figure 2b). On the contrary to the nickel-alloyed material, the titanium-containing alloy exhibited strong chipping wear (Figure 6c) in addition to the abrasive wear. The extensive chipping wear was probably caused by the presence of brittle (Fe,Ti)_5_Si_3_ particles, as confirmed by the morphology and size of these particles (Figure 2c).

### 3.3. High-Temperature Oxidation

**Figure 7:**
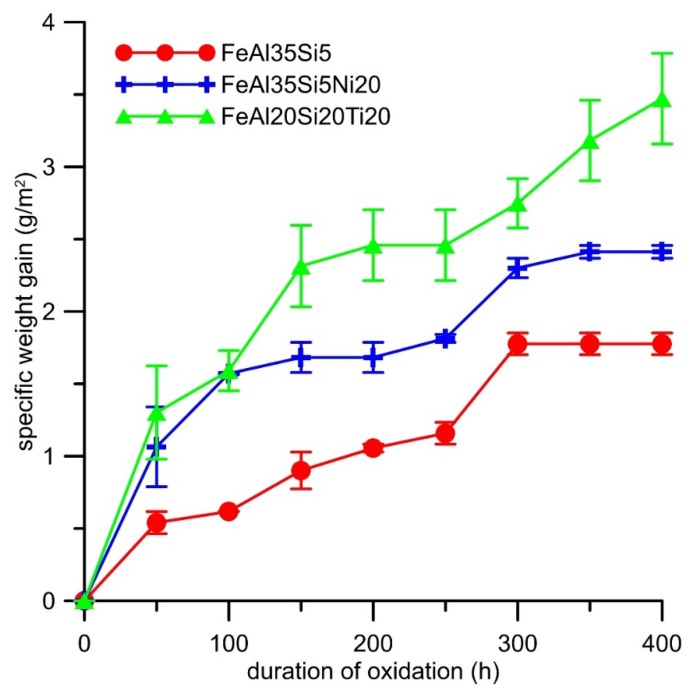
Dependence of specific weight gain (g∙m^−2^) on the duration of cyclic oxidation at 800 °C in air.

**Figure 8:**
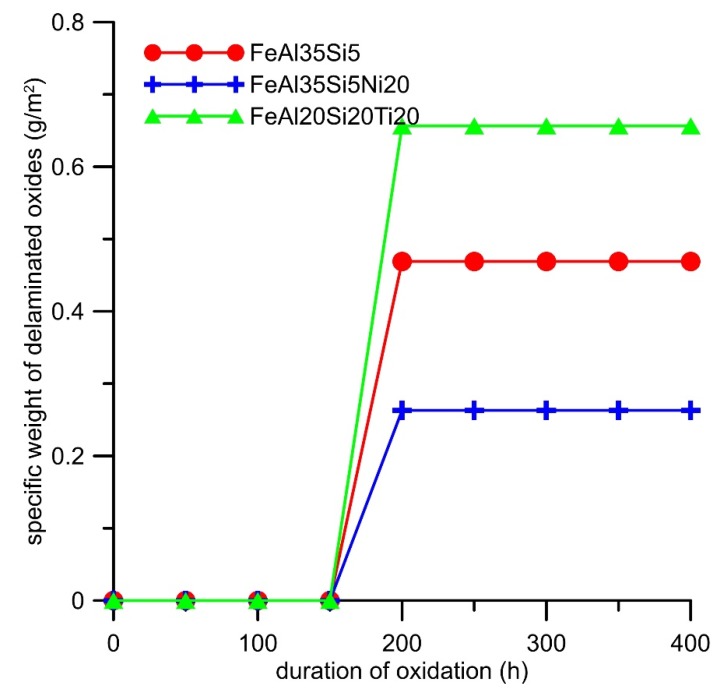
Dependence of the weight of delaminated oxides (g∙m^−2^) on the duration of cyclic oxidation at 800 °C in air.

Cyclic oxidation tests revealed that both alloying elements increased the specific weight gain due to the oxidation at 800 °C in the air (Figure 7). The oxidation kinetics of all alloys almost followed a parabolic dependence (Figure 7). The effect of titanium on the oxidation resistance was more detrimental than that of nickel. The dependencies were fitted by the parabolic law, and the resulting parabolic rate constants are presented in Table 4. The spallation of oxides was observed from the time of 200 h for all alloys (Figure 8). The titanium addition increased the amounts of delaminated oxides, while nickel lowered it; see Figure 8.

**Figure 9:**
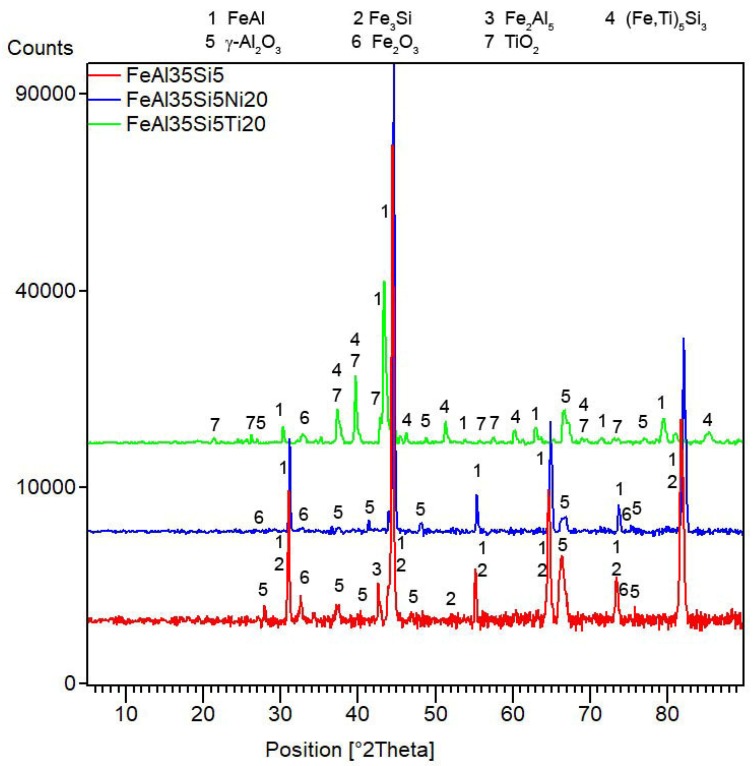
XRD patterns of the tested bulk alloys after cyclic oxidation at 800 °C for 400 h in air.

**Figure 10:**
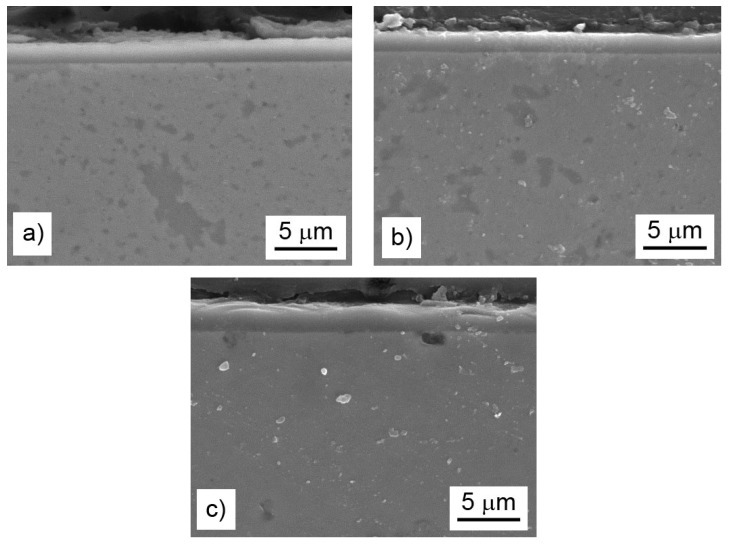
Microstructure (secondary electrons (SE)-SEM) of the oxide layers on the bulk alloys after cyclic oxidation at 800 °C for 400 h in air: the FeAl35Si5 alloy (**a**), the FeAl35Si5Ni20 alloy (**b**), and the FeAl35Si5Ti20 alloy (**c**).

The difference in the oxidation behavior of the titanium- and nickel-alloyed materials was probably given by the phase composition and microstructure of the oxide layers. The analyses confirmed that the main oxidation product of all tested materials was γ-Al_2_O_3_ (Figure 9). The other constituents differed based on the chemical composition of the alloys. While nickel almost did not participate in the formation of the oxide layer (Table 5), titanium formed TiO_2_ (rutile, P4_2_/mnm); see Figure 9. Rutile is known to be porous and non-adherent to a material when formed as an oxidation product at high temperatures, and this fact was also reflected in the microstructure of the oxide layers and their spallation behavior (Figure 8). The oxide layers formed on the FeAl35Si5 and FeAl35Si5Ni20 alloys were dense and compact (Figure 10a,b), but the oxide scales on the titanium-containing FeAl35Si5Ti20 alloy were less uniform, exhibiting a visibly higher surface roughness (Figure 10c).

## 4. Discussion

The results presented above provide the characterization of innovated Fe-Al-Si alloys with lower amounts of silicon. Regarding the production route, technology consisting of mechanical alloying and spark plasma sintering was selected. In this study, laboratory equipment was used, only allowing for the production smaller samples. However, the devices for MA and SPS, which are at the market, allowed for the manufacturing of products up to approximately 200 mm in diameter, which could really serve as a semi-product for many smaller parts, such as for exhaust valves of a combustion engine and other thermally loaded parts. In addition, a high-throughput SPS device for the series net-shape production of small parts is also already commercially available [26]. Compared to our previously-tested grade based on an alloy containing 60 wt. % of iron, 20 wt. % of aluminum, and 20 wt. % of silicon (designated as FeAl20Si20), these alloys contained more aluminum (35 wt. %) and less silicon (5 wt. %). The motivation for these tests was the enormously high room-temperature brittleness of the FeAl20Si20 alloy. At high temperatures, the toughness of the alloy was much better, and the alloy even exhibited a limited plasticity [16]. On the other hand, the FeAl20Si20 alloy did not resist sudden changes of temperature, which was already visible during cooling from the SPS process temperature. To prevent the formation of cracks, a slow cooling regime had to be employed. It was also visible during the wear resistance tests. Since the wear test was carried out without any lubricant and the alumina ball was unable to conduct the heat from the wear track, the temperature was allowed to rise locally. As a result of consequent cooling, cracks were visible in the wear track [27]. Such cracks were completely absent in the FeAl35Si5 alloy and its derivates; see Figure 6. The reason why these innovated alloys resisted the thermal shocks probably lies in the phase composition of the alloys. The previously-investigated high-silicon alloys are very heterogeneous because they are composed of Fe_3_Si, FeSi and Fe_3_Al_2_Si_3_ phases. Even though the silicides are relatively brittle, the most probable cause and the initiator of the cracks was Fe_3_Al_2_Si_3_, which was detected to be the continuous phase in the FeAl20Si20 alloy. On the other hand, the alloys that were tested in this work were based on iron aluminide as the matrix phase, which is considerably less brittle than Fe_3_Al_2_Si_3_. The alloying elements (Si, Ni, and Ti) made the alloys much harder than the usual hardness values of iron aluminide [2]. Even though the values of the wear rate were a bit higher for these innovated lower-silicon alloys (3 × 10^−6^ and 10 × 10^−6^ or higher for the FeAl20Si20- and FeAl35Si5-based alloys, respectively), they were still comparable with common tool steels [23], and they were able to be so without any risk of thermally-initiated cracks in the worn material.

The hardness values of all the materials tested in this work, which were based on an iron aluminide matrix, were considerably higher than the reported hardness of the B2 FeAl phase [2]. There are two reasons for this. The first one is the refinement of the structure by mechanical alloying, which caused Hall–Petch strengthening by the pinning of the slip dislocations by the grain boundaries. The fine structure was confirmed by XRD; see Table 2. The second effect, which was proven in this work, is the strengthening by silicon and the addition of the other alloying elements. Silicon formed fine particles of the Fe_3_Si phase, which could have had a reinforcing effect but also caused the changes in the lattice of the FeAl phase. The measured interplanar distance of the (110) planes (the most intense diffraction line) in the FeAl phase in the FeAl35Si5 alloy was 2.04024 × 10^−10^ m, while the tabulated one for the pure FeAl phase was 2.05697 × 10^−10^ m. This shows that the FeAl phase was more closely packed when the aluminum was partially substituted by silicon. The reason for this change was the atomic radius of silicon (111 pm), which is slightly lower than that of aluminum (118 pm). The other applied alloying elements, i.e., nickel and titanium, also influenced this distance. Nickel further decreased the (110) interplanar distance because it substituted iron and has a lower atomic radius (149 and 156 pm for nickel and iron, respectively). On the other hand, titanium increased the interplanar distance of the (110) planes in FeAl, because it has a much higher atomic radius (176 pm) than iron, which was expected to be substituted. In addition, titanium also reinforced the material through the formation of the (Fe,Ti)_5_Si_3_ silicide. However, this brittle phase detached during the wear test. It also caused the wear rate to increase in a way that was not fully proportional to the friction coefficient, i.e., the wear rate increased more than the friction coefficient; see Table 3. It can be concluded that it is more suitable to have a harder homogeneous solution-strengthened material to have a high wear resistance, like in the case of the nickel-containing alloy.

The effect of silicon on the oxidation behavior was thoroughly discussed in our previous works [15,17]. The effect of alloying elements (Ni and Ti) on the oxidation behavior was examined in this work. It was found that nickel almost did not participate on the formation of the oxide layer (Table 5). The reason for this is the fact that nickel oxide (NiO) has a higher value of the Gibbs energy of formation than iron oxide (Fe_2_O_3_) and a much higher value of the Gibbs energy of formation than aluminum oxide (Al_2_O_3_) (Table 6), which was the main constituent of the oxide layer. This implies that nickel would not tend to oxidize in this alloy. Additionally, given that mixtures of iron oxide and silicon oxide were previously found to be the high-temperature oxidation products of the Fe_3_Si phase [17], the elimination of iron silicide by the addition of nickel caused the minimization of the amount of iron in the oxide layer (Table 5). The Pilling–Bedworth ratio (the molar volume of the oxide divided by the molar volume of the material) that was calculated for the combination Al_2_O_3_/FeAl was approximately 1.8. The addition of iron increased this ratio due to the higher molar volume of the iron oxide. Therefore, the iron oxide lowered the adherence of the oxide layer to the material [17], so the lowering of its amount in the oxide layer resulted in the lowering of the amount of delaminated oxides (Figure 8). On the contrary, titanium has much higher stability of oxide, i.e., lower Gibbs energy of its formation, than iron (Table 6), and it probably came to the oxide layer after the zone below the oxide layer became strongly depleted by aluminum, which is more prone to oxidation. This phenomenon of depletion by aluminum was described in our recent paper [15]. Titanium dioxide, at the temperature of the oxidation test that formed in the rutile modification, is known to be very porous component of oxide layers with almost no protective effect [20]. The reason for this is the low molar volume of rutile, which caused a low Pilling–Bedworth ratio and thus the non-compact oxide scales. For this reason, the titanium-containing alloy oxidized more rapidly than the other tested alloys, and the delamination of the oxides was the highest.

## 5. Conclusions

In this work, the effects of nickel and titanium on the properties, particularly hardness, wear resistance and high-temperature oxidation resistance, of an FeAl35Si5 alloy were studied. It was shown that both of the alloying elements increased the hardness. In the case of nickel, the hardness increase was caused by solution strengthening of the aluminide phase, while the titanium addition reinforced the material through the formation of a titanium-containing silicide. Nickel had a positive effect on the wear resistance, while the influence of titanium was detrimental. Nickel lowered the likelihood of the delamination of the oxide layer during high-temperature oxidation. On the other hand, the effect of titanium was the opposite. From the viewpoint of the tested properties, the addition of nickel to the FeAl35Si5 alloy could be recommended.

## Figures and Tables

**Table 1:** Nominal chemical composition of the tested alloys.

Alloy Designation	Percentage by Weight (wt. %)				
	Fe	Al	Si	Ni	Ti
FeAl35Si5	40	35	5	0	0
FeAl35Si5Ni20	40	35	5	20	0
FeAl35Si5Ti20	40	35	5	0	20

**Table 2:** Interplanar distance of the (110) planes in the FeAl phase and the crystallite size of the FeAl phase in tested alloys.

Alloy Designation	d(110) of FeAl (× 10^−10^ m)	Crystallite Size of FeAl (× 10^−10^ m)
FeAl35Si5	2.0402	293
FeAl35Si5Ni20	2.0329	396
FeAl35Si5Ti20	2.0863	314

**Table 3:** Hardness, wear rate and friction coefficient of tested bulk alloys.

Alloy Designation	Hardness (HV 1)	Wear Rate (× 10^−6^ mm^3^∙N^−1^∙m^−1^)	Friction Coefficient (-)
FeAl35Si5	819 ± 20	18.3 ± 0.6	0.567
FeAl35Si5Ni20	914 ± 19	10.0 ± 0.3	0.365
FeAl35Si5Ti20	963 ± 13	63.7 ± 1.5	0.667

**Table 4:** Calculated parabolic rate constants of the cyclic oxidation of the tested alloys.

Alloy Designation	Parabolic Rate Constant (× 10^−6^∙g^2^∙m^−4^∙s^−1^)
FeAl35Si5	1.86
FeAl35Si5Ni20	4.94
FeAl35Si5Ti20	8.11

**Table 5:** Chemical composition of the oxide layers (EDS).

Alloy Designation	Percentage by Weight (wt. %)					
	Al	O	Fe	Si	Ni	Ti
FeAl35Si5	52.3 ± 2.4	39.1 ± 0.7	8.1 ± 1.8	0.5 ± 0.2	-	-
FeAl35Si5Ni20	49.3 ± 0.6	45.5 ± 0.8	3.8 ± 0.7	1.0 ± 0.3	0.4 ± 0.2	-
FeAl35Si5Ti20	41.6 ± 1.2	42.7 ± 1.9	6.3 ± 2.0	4.0 ± 0.8	-	5.4 ± 1.2

**Table 6:** Gibbs energy of formation (ΔG_f_) of selected oxides at 800 °C recalculated to one mol of oxidized metal [28,29].

Oxide Formula	ΔG_f_(800 °C) (kJ∙mol^−1^)
Al_2_O_3_	−889
Fe_2_O_3_	−491
NiO	−140
TiO_2_	−750
